# Supplementary material for: Reduced dorsal CA1 activity limits retention of the temporal component of declarative memory in the Cntnap2 knockout mouse model of autism
Source: eLife. 2026 Oct 13;15:RP111851. doi: 10.7554/eLife.111851 (PMC13661045; doi:10.7554/eLife.111851)
Supplement: Peer review history

# Editor report: eLife Assessment

**DOI:** 10.7554/eLife.111851.3.sa0

**Authors:** Laura A Bradfield

**Affiliations:** The University of Sydney Australia

**Keywords:** Convincing, Important

This **important** study uses the *Cntnap2* mouse model of autism to investigate how reduced activity in the dorsal CA1 region of the hippocampus contributes to impaired temporal binding and memory flexibility. The authors combined trace conditioning and radial maze assays with fibre photometry, optogenetic rescue, and brain-wide c-Fos mapping to provide **convincing** evidence that dorsal CA1 hypoactivity limits the retention of temporally separated associations and biases learning towards less flexible strategies. The work advances understanding of hippocampal contributions to cognitive alterations associated with autism and will be of broad interest to researchers studying memory, hippocampal function, and neurodevelopmental disorders.

# Referee report: Reviewer #2 (Public review)

**DOI:** 10.7554/eLife.111851.3.sa1

The authors investigate the contribution of dorsal CA1 hippocampal dysfunction to cognitive impairments in the *Cntnap2* knockout mouse model of autism spectrum disorder. Using two complementary behavioral paradigms, trace fear conditioning and a relational/declarative memory radial maze task, together with fiber photometry, optogenetic manipulation, and cFos mapping, they examine whether altered CA1 function contributes to deficits in temporal binding and memory flexibility.

A major strength of the study is the combination of behavioral, recording, and causal manipulation approaches. The trace fear conditioning experiments show that *Cntnap2* knockout mice retain associations across shorter temporal intervals but fail when the temporal gap is increased to 40 s. Fiber photometry reveals reduced dorsal CA1 activity under these conditions, and, importantly, optogenetic activation of dorsal CA1 pyramidal neurons during the trace interval rescues subsequent memory performance. This provides compelling evidence for a causal contribution of dorsal CA1 activity to the temporal binding deficit observed in this model.

The radial maze experiments extend these findings to a more complex form of relational/declarative memory. *Cntnap2* knockout mice are able to acquire the task but show impaired flexibility when previously learned spatial relations must be recombined. Their behavior is also characterized by greater lateralization, consistent with increased reliance on an egocentric rather than an allocentric spatial strategy. The revised manuscript now explains more clearly how this paradigm distinguishes relational/declarative from procedural learning strategies and how the 20-s inter-trial interval introduces a temporal binding requirement. This clarification substantially improves the conceptual link between the two behavioral paradigms.

The accompanying cFos analyses further show reduced recruitment of hippocampal regions and increased engagement of striatal and prefrontal regions in *Cntnap2* knockout mice. These findings are consistent with altered recruitment of memory systems accompanying the behavioral strategy shift. However, unlike the optogenetic experiments in the trace fear conditioning paradigm, the cFos measurements remain correlational. They therefore support, but do not by themselves establish, a causal reorganization from hippocampal-dependent declarative memory toward striatum-dependent procedural learning. The authors have appropriately moderated this interpretation in much of the revised manuscript, although some statements, particularly in the Abstract, could still be phrased more cautiously.

The revised manuscript also addresses the potential influence of the well-described hyperactivity of *Cntnap2* knockout mice. The distinction between hyperactivity and impulsive-like behavior is now more explicitly discussed. In the trace fear conditioning experiments, comparable resting and freezing behavior under control conditions argues against locomotor activity accounting for the memory phenotype. In the radial maze, shorter decision latencies combined with longer post-choice running times are more consistent with reduced deliberation than with a simple increase in locomotor activity. Importantly, the authors acknowledge that the task was not specifically designed to measure impulsivity.

Overall, the revision has strengthened the manuscript and addressed my main concerns. The principal conclusion-that reduced dorsal CA1 activity contributes causally to impaired temporal binding in *Cntnap2* knockout mice-is well supported by the converging behavioral, photometric, and optogenetic evidence. The broader proposal that hippocampal dysfunction is accompanied by greater reliance on egocentric/striatal learning strategies is also supported by the behavioral and cFos data, provided that it is interpreted as an association rather than a demonstrated causal reorganization of memory systems.

The study makes a valuable contribution by linking hippocampal circuit dysfunction to specific components of declarative memory in a widely used model of autism. More broadly, the combination of temporal binding and memory flexibility paradigms provides a useful framework for investigating how hippocampal dysfunction may alter the organization and flexible expression of memory in neurodevelopmental disorders.

# Referee report: Reviewer #3 (Public review)

**DOI:** 10.7554/eLife.111851.3.sa2

Summary:

The manuscript evaluated behavioral phenotypes in the *Cntnap2* knockout mouse using two behavioral paradigms: trace fear conditioning and a radial maze task. The trace fear conditioning training is normal, but memory generalization is impaired. The inflexibility is suggested to be related to low activity in dCA1 neurons, which can be rescued by ChR2. The radial maze task data suggested a similar conclusion. Brain-wide cFos mapping indicated impairments in the *Cntnap2* knockout mouse. The brain-wide cFos mapping does not show direct correlations with *Cntnap2*, limiting the interpretation of these data in the context of this paper.

Strengths:

The behavior data are solid.

Comment on revised version.

The authors have addressed all my concerns.

# Author comment: Author response

**DOI:** 10.7554/eLife.111851.3.sa3

**Authors:** Elise Caitlin Rawlinson, Michael H McCullough, Azza Sellami, Aline Marighetto, Alice Shaam Al-Abed, Nathalie Dehorter

**Affiliations:** Australian National University Acton Australia; The University of Queensland Brisbane Australia; Neurocentre Magendie, Université de Bordeaux Bordeaux France; Neurocentre Magendie, Université de Bordeaux Bordeaux France; Australian National University Acton Australia; University of Queensland and Australian National University Brisbane Australia

The following is the authors’ response to the original reviews.

**Public Reviews:**

**Reviewer #1 (Public review):**

Summary:

The uniqueness of this paper is the study of the formation of temporal binding-dependent memories in the cntnap2 mouse, a long-standing mouse model of autism that has been used to test therapeutic modalities.

Strengths:

I liked the combination of optical recordings and interventions and the backup of primary observations with control experiments.

Weaknesses:

(1) Fiber photometry recordings are too coarse to give salient clues to the underlying mechanism.

We acknowledge that fiber photometry provides population-level measurements and does not resolve the activity of individual neurons or synaptic mechanisms. Our aim was to identify alterations in the activity of defined neuronal populations during behaviour rather than to delineate the underlying cellular mechanisms. We agree that future studies employing higher-resolution approaches, such as two-photon calcium imaging, in vivo electrophysiology, or single-cell recordings, would provide important mechanistic insights into the circuit changes underlying the observed activity patterns.

(2) Are perturbed pyramidal cells causally responsible for the altered trace? What can be concluded about the possible role of inhibitory interneurons as potential drivers? The observations focus on abnormal regional activity as observed with fiber photometry and manipulated by optogenetics. The authors should state clearly the limits of their conclusions.

Our optogenetic experiments demonstrate a causal role for the targeted neuronal population in modulating the observed activity and behavioural phenotype. However, we do not conclude that this population is solely responsible for generating the altered fiber photometry signal, nor do we infer that it represents the exclusive driver of the underlying circuit dysfunction. Rather, our findings demonstrate that manipulating this population is sufficient to alter circuit activity, while acknowledging that the recorded signals likely reflect interactions between multiple neuronal populations. We have revised the manuscript to make these distinctions clearer.

(3) I found the "trace" nomenclature confusing. "....in which mice are required to memorize the association between a tone (Conditioned Stimulus) and a mild electric foot-shock (Unconditioned Stimulus), separated by a time interval called Trace (Sellami et al., 2017)." It seems that the conceptual model invokes the creation of an [eligibility] trace, characterized by its progressive disappearance over time. It may be a convention in the field or a matter of language, but it seems perverse to use "trace" to label the time interval rather than the entity that is decaying. If this is an accepted convention going back to Howard Eichenbaum, the authors should cite the paper that first introduced the convention.

We thank the reviewer for this comment. This is indeed an established convention in the classical/Pavlovian conditioning literature rather than terminology specific to our study or to Sellami et al. (2017). The term "trace conditioning" was coined by Pavlov (1927), who used "trace interval" to designate the empty period separating CS offset from US onset, precisely because — as the reviewer intuits — successful conditioning across this gap requires the organism to maintain a memory trace of the CS. In other words, the interval is named for the cognitive/neural entity (the decaying CS trace) that must be sustained across it, not because the interval itself is thought to be a physical or decaying object. So "trace interval" is shorthand for "the interval across which a trace must be maintained," analogous to how "delay conditioning" refers to a paradigm named for a temporal property of the procedure rather than the mechanism per se. We have added a citation to Pavlov (1927) at first use of the term, and clarified the phrasing to make the etymology explicit, as suggested.

(4) I would advocate for the addition of some discussion points for the authors to consider.

(a) Is the retention of activity in CA1 related to phenomena at the cellular or subcellular level in CA1 pyramidal cells? I'm thinking of dendritic, delayed, and stochastic CaMKII activation (DDSC) as defined by Yasuda's group or short-term and associative plasticity of calcium dynamics (STAPCD) as delineated by Caya-Bissonette and Beique.

We thank the reviewer for this insightful suggestion. Our study was designed to investigate network-level dynamics, and the approaches used do not allow us to determine whether the retained activity in CA1 arises from intracellular mechanisms, such as dendritic calcium dynamics or CaMKII-dependent signalling (including mechanisms such as DDSC or STAPCD), recurrent circuit interactions, or a combination of both. We therefore cannot directly assess the contribution of these cellular and subcellular processes. We have now added a paragraph to the Discussion acknowledging that persistent dendritic calcium signalling and CaMKII-dependent plasticity are plausible contributors to sustained CA1 activity and represent an important avenue for future investigation.

(b) Was the optogenetic intervention ever administered in a delayed fashion, capitalizing on the temporal advantages of optogenetics to probe dynamics?

This is indeed an important control. While we did not perform this intervention in the current study, this control was part of our seminal study demonstrating the causal role of the dCA1 in temporal binding (Sellami et al., PNAS, 2017, Fig. 1E doi: https://doi.org/10.1073/pnas.161965711). We showed that aged mice had reduced temporal binding capacity, associated with decreased dCA1 activity. We successfully rescued temporal binding capacity in aged mice through ChR2-induced activation of dCA1 pyramidal neurons during the trace interval, but not outside of the trace interval. Given the striking similarity between the temporal binding deficits observed in aged mice and those reported here in *Cntnap2* KO mice, we did not repeat this previously established control in the present study. To acknowledge this point, we have also added a statement to the Discussion noting that confirming the temporal specificity of dCA1 optogenetic manipulation in the *Cntnap2* KO model will be an important direction for future studies.

(c) Is the newfound reliance on corticostriatal pathways something more than compensation at the behavioral level? Could it be driven in part by the ASD-related genetic changes?

We agree that the increased reliance on corticostriatal pathways could reflect both compensatory recruitment at the behavioral level and a direct consequence of ASD-related genetic alterations affecting circuit development and function. Our current data demonstrate a shift in circuit engagement but do not allow us to distinguish whether this represents an adaptive compensation or a primary consequence of *Cntnap2* deficiency. However, given that ASD-associated mutations can alter the development, connectivity, and plasticity of corticostriatal circuits, it is possible that the observed changes reflect intrinsic circuit reorganization rather than solely a compensatory behavioral strategy. We have revised the Discussion to acknowledge this possibility and to clarify that altered corticostriatal recruitment may represent a direct consequence of the genetic disruption that subsequently shapes behavioral strategies.

**Reviewer #2 (Public review):**

The authors investigate the contribution of dorsal CA1 hippocampal dysfunction to cognitive impairments in the *Cntnap2* knockout mouse model of autism spectrum disorder. Building on previous evidence implicating the hippocampus in episodic and relational memory processes, they combine trace fear conditioning, fiber photometry, optogenetic manipulation, a relational/declarative memory radial maze task, and cFos mapping to test whether altered CA1 function contributes to deficits in temporal binding and memory flexibility.

The study has several important strengths. First, the work addresses a relatively understudied aspect of autism-related cognition, namely hippocampal-dependent memory processes, whereas much of the literature has focused on social behavior, cortical circuits, or striatal dysfunction. Second, the authors employ multiple complementary approaches that converge on a coherent mechanistic hypothesis. The behavioral data demonstrate a reduced ability of *Cntnap2* knockout mice to retain associations across long temporal gaps. Fiber photometry recordings reveal reduced dorsal CA1 activity during conditions that challenge temporal binding, and optogenetic activation of dorsal CA1 neurons during the trace interval is sufficient to rescue memory performance. Together, these findings provide strong support for a causal contribution of dorsal CA1 activity to temporal binding deficits in this model.

The second major strength of the manuscript is the extension of these findings to a more complex hippocampus-dependent memory task. The radial maze experiments indicate that *Cntnap2* knockout mice show impaired memory flexibility and a greater reliance on egocentric learning strategies. The accompanying cFos analyses suggest altered recruitment of hippocampal and striatal networks during learning, providing a systems-level framework that may explain the observed behavioral phenotype.

Overall, the main conclusions regarding impaired temporal binding and reduced dorsal CA1 engagement are well supported by the data. The optogenetic rescue experiments are particularly compelling because they move beyond correlation and directly test causality. The manuscript therefore makes a meaningful contribution to our understanding of how hippocampal dysfunction may contribute to cognitive abnormalities associated with autism.

Weaknesses:

Some conclusions are necessarily more inferential than others. In particular, the interpretation that the observed behavioral phenotype reflects a broader shift from hippocampal-dependent declarative memory toward striatum-dependent procedural learning is supported primarily by cFos activity patterns and behavioral strategy measures. While the data are consistent with this interpretation, they do not directly demonstrate a causal reorganization of memory systems. Similarly, although the findings identify a mechanism in the *Cntnap2* model, caution is warranted when extrapolating these conclusions to autism spectrum disorder more broadly; but I believe this caution is addressed in the discussion.

We agree that our data do not directly demonstrate a causal reorganization of memory systems with cFOS activity patterns. Our intention was to propose that the behavioural strategy together with the brain-wide cFos activation patterns are consistent with a shift in the relative engagement of hippocampal- and striatal-dependent networks. We have revised the manuscript to moderate our interpretation throughout, replacing causal language with wording that reflects an association between the observed behavioural changes and altered recruitment of these memory-related circuits.

Despite these limitations, the study presents a coherent and well-executed body of work that provides novel mechanistic insight into hippocampal contributions to cognitive dysfunction in a widely used autism model. The findings should be of considerable interest to researchers studying hippocampal function, memory systems, and neurodevelopmental disorders.

**Reviewer #3 (Public review):**

Summary:

The manuscript evaluated behavioral phenotypes in the *Cntnap2* knockout mouse using two behavioral paradigms: trace fear conditioning and a radial maze task. The trace fear conditioning training is normal, but memory generalization is impaired. The inflexibility is suggested to be related to low activity in dCA1 neurons, which can be rescued by ChR2. The radial maze task data suggested a similar conclusion. Brain-wide cFos mapping indicated impairments in the *Cntnap2* knockout mouse. The brain-wide cFos mapping does not show direct correlations with *Cntnap2*, limiting the interpretation of these data in the context of this paper.

We agree that brain-wide cFos mapping does not directly identify the molecular or cellular mechanisms by which *Cntnap2* deficiency alters circuit function. Rather, we use cFos as a functional readout of network recruitment during behaviour. Our interpretation is therefore limited to identifying differences in activity patterns associated with the behavioural phenotype, rather than establishing direct mechanistic links to *Cntnap2* function. We have clarified this point in the revised manuscript and moderated the text accordingly.

Strengths:

The behavior data are solid.

Weaknesses:

The underlying mechanism is not fully investigated.

Major points：

(1) The authors should thoroughly check their manuscript as there are many typos in the current version that affect the readability.

(2) In trace fear conditioning, the tone test impairment can be rescued by ChR2. Have the authors tried rescue experiments with *Cntnap2*? Rescue experiments in the radial maze task are also essential, either with ChR2 or *Cntnap2*.

We agree that rescue experiments in the radial maze would provide additional mechanistic insight by testing whether restoring dCA1 activity optogenetically in *Cntnap2* KO mice is sufficient to rescue declarative memory flexibility. However, we have already established the causal role of dCA1 in temporal binding and relational/declarative memory in the radial maze task in our earlier work (Sellami et al., PNAS, 2017, Figure 2E). Indeed, optogenetic inhibition of dCA1 in the radial maze task, specifically during the inter-trial interval, prevented flexible relational/declarative memory formation. In the present study, we confirmed the causal contribution of dCA1 activity to temporal binding in *Cntnap2* KO mice in the trace fear conditioning paradigm (Figure 1J-L). Therefore, repeating the optogenetic experiment in the radial maze would provide only limited additional information, while requiring dedicated experimental cohorts and additional validation. Moreover, as the team is based in 2 different institutions and countries (France and Australia), we unfortunately do not have the capacity to perform these experiments.

Regarding a rescue with the *Cntnap2* protein, here, we used the *Cntnap2* KO as a well-accepted model of autism spectrum disorder (ASD), rather than understanding the role of this protein in temporal binding capacity and memory formation. The present study was hence designed to determine how dCA1 activity relates to temporal binding and memory performance in ASD, building on the causal evidence established in our previous work. We therefore consider *Cntnap2*-specific rescue experiments an important follow-up to further test the sufficiency of dCA1 activation, rather than an essential experiment for establishing the conclusions of the present study.

We have highlighted these points as an important direction for future investigations and revised the manuscript to better distinguish our findings from this additional mechanistic question.

(3) The quality of the cFos example image in Figure 3 is too low. The authors should also provide example images for the other brain regions in the supplementary data, if possible.

The low quality of the cFos image in Figure 3 resulted from a formatting issue during figure uploading. This has now been corrected, and a higher-resolution image has been included in the revised manuscript. We have also added representative images for the other brain regions analyzed to the Supplementary Information, as requested.

(4) The causal link between the brain-wide cFos mapping and the *Cntnap2* knockout is weak. How to explain the increase of cFos cell densities in some brain regions, but the decrease in others?

We agree that cFos mapping cannot explain the mechanisms underlying the regional increases and decreases in activity. We interpret these bidirectional changes as reflecting differential recruitment of distributed brain networks rather than direct effects of *Cntnap2* deficiency on individual brain regions, and have clarified this in the revised manuscript.

**Recommendations for the authors:**

**Reviewer #1 (Recommendations for the authors):**

(1) Improve clarity of the Figure layout: Figure 1, panel I legend. I think the identification of experimental groups is in the wrong place. It really applies to panels K, N, and L, not to panel I. This is important because the genetic stimulation in panels K, N, and L is the key experiment in this whole figure.

We agree that the placement of the experimental group identification in the original figure legend could be confusing. We have revised the figure layout and legend so that the description of the experimental groups is now associated with panels K and L, where the optogenetic stimulation experiments are presented, thereby improving the clarity of the figure.

(2) Fix Figure references. "This indicates impaired retention of trace fear memory and temporal binding ability, relative to WT mice (Figure 2I)....: [there is no Fig. 2I, so I'm guessing you mean Fig. 1I; subsequent Figure references also seem to confuse Fig. 1 and 2].

We thank the reviewer for identifying these errors. We have carefully checked and corrected all figure citations throughout the manuscript, including the reference to Figure 1I-L in this section, to ensure that each citation now refers to the appropriate panel.

(3) Missing words? In both T20 and T40 conditions, regardless of genotype, [the response to] tone frequency significantly decreased.

We thank the reviewer for spotting this omission. The sentence has been corrected to read: "In both T20 and T40 conditions, regardless of genotype, the response to tone frequency significantly decreased."

(4) Jargon that should be declared at an appropriately early point. R/DM = relational/declarative memory.

We have now introduced the term as relational/declarative memory (R/DM) at its first appearance in the manuscript and have ensured that the abbreviation is used consistently thereafter.

(5) Confusing statement? "In the 40s trace-conditioned group, while the frequency of the calcium transients did not differ between WT and *Cntnap2* KO mice throughout conditioning (Figure S1G), their amplitude was significantly reduced both during the presentation of the second tone and consistently across the three trace intervals in *Cntnap2* KO mice (Figure 1I)."

We have revised this sentence to improve its clarity and explicitly distinguish between the frequency and amplitude of calcium transients. The revised text now makes clear that, although the frequency of calcium transients did not differ between genotypes throughout conditioning, their amplitude was significantly reduced in *Cntnap2* KO mice during the second tone presentation and across the three trace intervals.

**Reviewer #2 (Recommendations for the authors):**

(1) The manuscript would benefit from a clearer distinction between conclusions directly supported by the data and broader interpretations. In particular, statements suggesting a shift from declarative to procedural memory systems could be presented more cautiously, as the cFos analyses provide indirect rather than causal evidence for such reorganization.

We thank the reviewer for this constructive comment. We have revised the manuscript throughout to more clearly distinguish conclusions that are directly supported by our data from broader interpretations. In particular, statements referring to a shift from declarative to procedural memory systems have been moderated.

(2) Additional clarification of the behavioral interpretation of the radial maze task would be valuable for readers who are less familiar with this paradigm, particularly regarding its relationship to relational/declarative memory and its distinction from procedural learning strategies. In addition, the results' interpretation in this paradigm is unclear to me. What is the significance of the 20-second delay in this task (why not use 10 sec or 60 sec)? I believe the paradigm tests spatial rather than temporal distant items (in contrast with trace fear conditioning that refers to temporally distant events)? Please explain further the link between the two behavioral paradigms.

We thank the reviewer for these comments.

Regarding the relationship between relational/declarative and procedural learning: The R/DM task was designed by our group (Marighetto and colleagues; Mingaud et al., 2007; Sellami et al., 2017, 2018) specifically to dissociate two learning/memory systems that can support the same behavioral output (correct arm choice) but that differ fundamentally in their underlying representations and, critically, in their flexibility.

During acquisition, an animal can solve each of the three arm-pair discriminations either by forming a flexible, relational representation of the whole spatial configuration (i.e. an allocentric/hippocampus-dependent "cognitive map" strategy, in which the reward's location is encoded relative to distal cues and to the other pairs) or by learning a set of rigid, response-based rules (i.e. an egocentric/striatum-dependent "turn left/turn right" procedural strategy tied to each specific pair).

Both strategies can produce equivalent accuracy during initial acquisition, which is why acquisition performance alone cannot distinguish them. The flexibility probe (recombining pairs A and B into a novel pair AB, without moving the reward) is the critical dissociation: only an animal that encoded the reward's location relationally, within a broader spatial map, can generalize correctly to this untrained configuration; an animal that relied on a rigid stimulus– response rule for each pair individually would be unable to solve the recombined pair above chance. Performance on pair AB is therefore the read-out of hippocampus-dependent relational/declarative memory, while the degree of left–right lateralization during acquisition (quantified by a lateralization index) is a converging behavioral signature of reliance on the egocentric/procedural system.

We have clarified this distinction in the Methods and Results to facilitate interpretation of the paradigm.

Regarding the 20-s delay and its relationship to trace fear conditioning: The reviewer is correct that the R/DM task and TFC differ in the information being integrated: the R/DM task involves spatially distinct discrimination episodes, whereas TFC involves temporally separated stimuli presented in the same context. However, both tasks require information separated by an interval to be integrated into a unified memory representation, a process referred to here as “temporal binding” (Sellami et al., 2017). The 20-s inter-trial interval in the R/DM task was based on our previous work, in which this interval was shown to be sensitive to age-related deficits in temporal binding and relational memory (Sellami et al., 2017). Similarly, our TFC experiments established a longer temporal-binding capacity in young mice (up to 40 s) compared with aged mice (20 s). Thus, although the two paradigms involve different types of information, they share the requirement to maintain and integrate information across a temporal gap. The different intervals used in the two paradigms reflect their distinct task structures and demands.

(3) Although the authors address potential locomotor confounds, a brief discussion of how hyperactivity and impulsivity might influence behavioral performance in the different paradigms would strengthen the interpretation of the results.

We thank the reviewer for raising this point. We agree that distinguishing hyperactivity from impulsivity strengthens the interpretation of our behavioral phenotypes, and we have added a discussion paragraph making this distinction explicit. Briefly: in the TFC paradigm, our existing open-field data (Figure S1D) show that while *Cntnap2* KO mice travel more distance and move faster, their resting time is unaffected relative to WT; so, the reduced freezing/temporal binding phenotype at the 40 s trace cannot be attributed to a general locomotor confound, since freezing (immobility) was actually comparable between genotypes during acquisition. In the R/DM radial maze task, however, we agree the phenotype is better read as impulsivity than hyperactivity *per se*: KO mice show shorter decision latencies specifically at the choice point (Figure 2G), with no corresponding genotype effect on their post-choice running speed for correct trials (Figure 2H), indicating that the effect is on deliberation before response rather than on general motor output. We have revised the Discussion to make this distinction, and its implications for interpreting the egocentric/procedural bias, explicit.

(4) Minor presentation issues:

- Ensure that all statistical tests, sample sizes, and post hoc comparisons are reported consistently throughout the manuscript and figure legends.

We have carefully reviewed the statistical reporting throughout the manuscript and figure legends to ensure consistency. We have clarified sample sizes, statistical tests, and post hoc comparisons where required and in the methods.

- Carefully proofread the manuscript for minor typographical and grammatical errors. For instance, (a) several figures numbers indicated in the main text do not correspond to the figures the authors refers to (page 5 Figure 2I, page 6 Figure 2J and 2K, page 7 "data in fig S2"... and so on); (b) rephrase (not clear) page 9: "Differences in the relative contribution of individual substructures between trained WT and *Cntnap2* KO mice involved dCA1, dCA2, and dCA3, and favored the DMS, IL, and ACC."

We thank the reviewer for highlighting these issues. We have carefully proofread the manuscript and corrected all figure reference errors and typographical inconsistencies throughout the text. We have also revised the unclear sentence on page 9 to improve clarity and accuracy.

- Page 9, an additional sentence is needed to explain the significance of the bibliographical reference: "Atypicalities in declarative memory have been reported in ASD, with specific impairments in relating items (Minor et al., 2023)."

We have revised this section to clarify the significance of the cited study and its relevance to our findings. Specifically, we have expanded the sentence to highlight that impairments in relational processing in ASD may reflect difficulties in integrating individual experiences into coherent memory representations, a process that relies on hippocampal function.

**Reviewer #3 (Recommendations for the authors):**

Major Points:

As stated in the public review, the authors need to thoroughly check their manuscript before submission. There are too many typos in the current version that affect the readability. For example, but not limited to:

(1) Page 5, "Figure 2I" should be "Figure 1I"; "Figure 2J" should be "Figure 1J"; "Figure 2K" should be "Figure 1K".

(2) The figure legends of Figure 1J-L are missing.

(3) Page 8, "In contrast, *Cntnap2* KO mice showed no learning-induced dCA1 activation together with hypoactivation of dCA3 in both naïve and trained groups compared to WT mice (Figure S2A)", should it be dCA2 instead of dCA1? Please double-check the manuscript.

We thank the reviewer for highlighting these issues. We have thoroughly checked the manuscript and corrected all typos, figure reference errors, and fixed figure legend information. Regarding the point (3), we did mean to describe the lack of training-induced activation of dCA1, but failed to reference back to Figure 3, likely causing the confusion. This has now been clarified. The manuscript has also been carefully proofread to improve clarity and readability.

Minor points:

There are two duplicate rows (two wt) in their raw datasheet, Fig3BCD & S2 and Fig3EF. Please correct them in case of further problems.

WT Naive 178.736 103.821 250.703 185.229 437.575 50.535 266.254 306.187 306.187 WT Naive 178.736 103.821 250.703 185.229 437.575 50.535 266.254 306.187 306.187

WT Naive 46.87 37.94 15.19 8.57 4.98 12.02 8.88 20.98 2.42 12.77 14.68 14.68 WT Naive 46.87 37.94 15.19 8.57 4.98 12.02 8.88 20.98 2.42 12.77 14.68 14.68

We thank the reviewer for noticing this mistake. These duplicated rows have been corrected.
